# Extensive Multicompartmental Air Dissection With Pneumoscrotum and Pneumorrhachis After Perineal Compressed‐Air Injury Without Demonstrable Persistent Full‐Thickness Rectal Perforation: A Case Report

**DOI:** 10.1002/ccr3.73291

**Published:** 2026-08-07

**Authors:** Van Trung Hoang, Vuong Hoang Trung Le, Vichit Chansomphou, The Huan Hoang

**Affiliations:** ^1^ Department of Radiology Thien Hanh Hospital Dak Lak Vietnam; ^2^ Department of Radiology Savannakhet Medical‐Diagnostic Center Kaysone Phomvihane Laos

**Keywords:** anorectal barotrauma, compressed‐air injury, computed tomography, multicompartmental air dissection, pneumorrhachis, pneumoscrotum

## Abstract

Anorectal barotrauma from compressed air is a rare but potentially life‐threatening injury, and most published cases involve frank colorectal perforation, whereas extensive extraluminal air dissemination without a demonstrable full‐thickness defect is poorly characterized. We report a previously healthy 21‐year‐old man who presented with acute anal pain after a coworker directed a high‐pressure air jet toward his perineum through two layers of clothing. He was hemodynamically stable, with preserved anal sphincter tone and no rectal bleeding, but had widespread subcutaneous crepitus over the chest and abdominal wall and a tense, distended scrotum. Computed tomography (CT) revealed extensive air within the retroperitoneum, perirenal spaces, abdominopelvic fascial planes, and subcutaneous tissues of the chest, abdomen, perineum, and scrotum, together with intraspinal air consistent with pneumorrhachis; no discrete persistent rectal wall defect, large fluid collection, or fecal contamination was identified. No water‐soluble rectal contrast leak study was performed; therefore, an occult sealed luminal communication could not be entirely excluded. Endoscopy at a tertiary referral center showed only superficial mucosal abrasion without a visible perforation, and the patient was managed conservatively with bowel rest, rectal lavage, and broad‐spectrum antibiotics, recovering uneventfully. These findings suggest that extensive air dissection may occur through a sub‐endoscopic mucosal breach or transiently sealed microperforation. CT was essential for mapping the air distribution and excluding high‐risk features; in a stable patient without peritonitis, fecal contamination, or clinical deterioration, this supported cautious conservative management.

## Key Clinical Message

High‐pressure perineal compressed‐air injury can cause extensive multicompartmental air dissection, including pneumorrhachis, even when CT and endoscopy show no full‐thickness rectal defect. Because an occult luminal communication cannot be excluded, CT should map air distribution and high‐risk features; conservative management suits stable patients without peritonitis or fecal contamination.

## 1 Introduction

Pneumatic injury of the colorectum from high‐pressure compressed air is uncommon but carries substantial morbidity, because a large volume of air can enter the anorectal canal within seconds. Such injuries occur almost exclusively in occupational settings, typically as a prank or during the careless cleaning of clothing with industrial air hoses [1, 2, 3]. Although direct insertion of the nozzle into the anus is the most widely recognized mechanism, colorectal barotrauma can also result from perineal blasting without direct insertion: a concentrated high‐pressure jet may overcome both clothing and anal sphincter resistance, particularly when the funnel‐shaped contour of the buttocks channels the airflow toward the anal canal [4, 5].

The clinical spectrum ranges from severe presentations with peritonitis, tension pneumoperitoneum, and hemodynamic compromise, which mandate urgent decompression and surgical exploration, to milder cases without overt perforation, in which the diagnosis depends primarily on cross‐sectional imaging [1, 2, 6]. Computed tomography (CT) is particularly informative, as it can map the precise distribution of extraluminal air along fascial planes and identify dissection into compartments that are otherwise clinically silent [7, 8].

Pneumorrhachis, the presence of free air within the spinal canal, is a rare radiologic finding that may be traumatic, iatrogenic, infectious, or spontaneous [9, 10]. It is most often discussed in the context of pneumomediastinum, thoracic trauma, or spinal procedures. However, air can theoretically reach the epidural space by tracking along contiguous retroperitoneal and paraspinal fascial planes and entering through the neural foramina [11, 12]. Extradural pneumorrhachis is usually benign and resolves with treatment of the underlying cause, whereas intradural air or accompanying neurological deficits raise concern for more severe pathology [10, 13].

We report a rare case of extensive multicompartmental air dissection, involving the retroperitoneum, subcutaneous tissues, scrotum, perineum, and spinal canal, after perineal compressed‐air injury, in which neither routine CT nor endoscopy demonstrated a persistent full‐thickness rectal wall defect. The case underscores the diagnostic value of CT and the possibility of conservative management even when the extraluminal air burden appears alarming.

## 2 Case Presentation

### 2.1 Clinical History and Physical Examination

A 21‐year‐old man with no relevant medical history presented to the emergency department of Thien Hanh Hospital with acute anal pain. Approximately 30 min earlier, while wearing underwear and shorts (combined estimated thickness ~1 cm), he had been unexpectedly exposed to a high‐pressure air jet directed toward his anus by a coworker. He developed immediate severe anal pain but denied chest pain, dyspnea, abdominal pain, vomiting, rectal bleeding, dysuria, hematuria, urinary retention, or testicular pain.

On arrival, he was alert, hemodynamically stable (heart rate 80 beats/min, blood pressure 110/70 mmHg), and showed no signs of respiratory distress. Digital rectal examination revealed a grossly intact anal canal, preserved sphincter tone, and no active bleeding or clot. However, palpable subcutaneous crepitus was detected over the anterior chest and abdominal wall, and the scrotum was bilaterally tense and distended, consistent with pneumoscrotum. The discordance between the unremarkable rectal examination and the widespread subcutaneous emphysema raised immediate concern for major air dissection.

### 2.2 Imaging Findings

**FIGURE 1:**
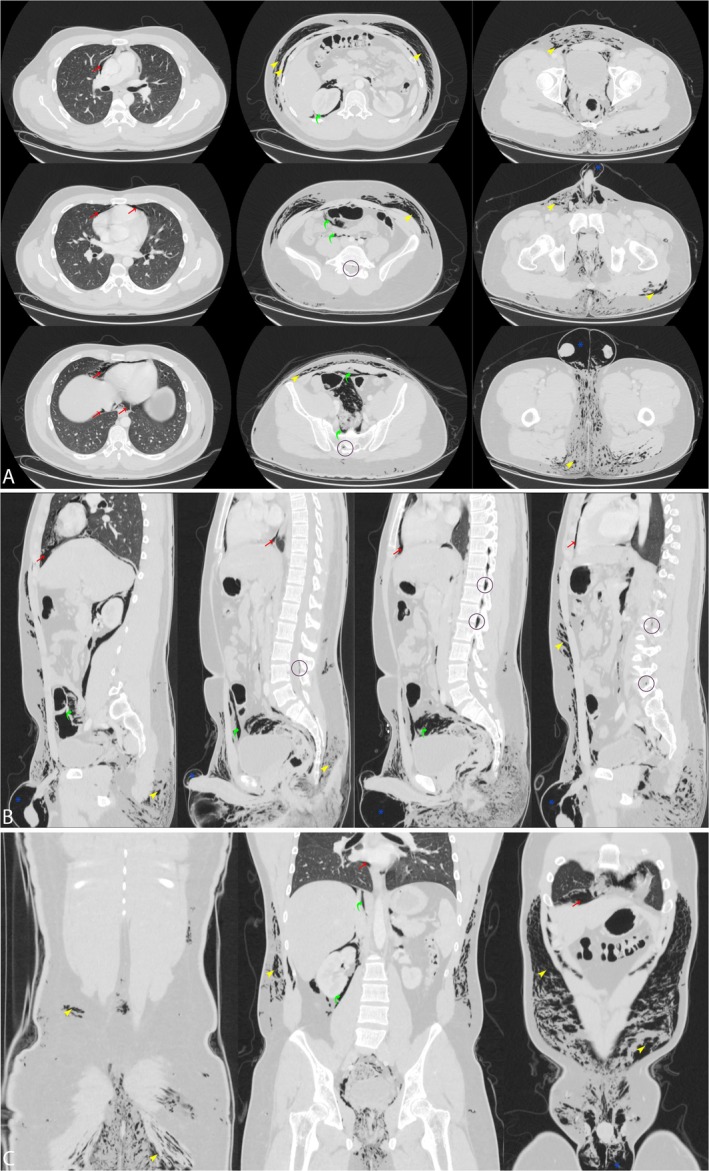
Computed tomography after perineal compressed‐air injury. Axial (A), sagittal (B), and coronal (C) images demonstrate extensive extraluminal air across multiple compartments. Air is present within the peritoneal and retroperitoneal spaces (green curved arrows), along the abdominopelvic fascial planes, and within the subcutaneous tissues of the chest wall, abdominal wall, and perineum (yellow arrowheads), as well as within the scrotum and penis (dark‐blue asterisks). Intraspinal air is also seen, consistent with pneumorrhachis, extending from approximately the T9 level caudally through the thoracolumbar and lumbosacral spinal canal, best appreciated on sagittal images (purple circles). Red arrows indicate air within the mediastinum, pericardial space, and bilateral pleural spaces. No definite persistent full‐thickness rectal wall defect or gross fecal contamination is evident on routine CT. The pattern is most consistent with widespread air dissection following an occult sub‐endoscopic anorectal mucosal breach, sealed luminal communication, or transient microperforation.

Emergency multidetector CT was performed in axial, sagittal, and coronal planes. The study demonstrated extensive extraluminal air distributed across multiple compartments (Figure 1). Air was present within the peritoneal and retroperitoneal spaces, prominently in the perirenal spaces, and tracked along the abdominal and pelvic fascial planes. Diffuse subcutaneous emphysema involved the chest wall, abdominal wall, perineum, and scrotum. Foci of intraspinal air were also identified, consistent with pneumorrhachis, and additional air was seen in the mediastinum, pericardial space, and bilateral pleural spaces. No discrete persistent rectal wall defect, large pelvic fluid collection, or fecal contamination was evident on the initial study. A water‐soluble rectal contrast enema or dedicated CT leak study was not performed before transfer; therefore, a transient or sealed luminal communication could not be definitively excluded. Overall, the imaging pattern was that of high‐pressure anorectal barotrauma with widespread air dissection, most likely arising from a sub‐endoscopic anorectal mucosal breach or transiently sealed microperforation rather than a demonstrable persistent full‐thickness tear.

### 2.3 Management and Outcome

Because of the extent of extraluminal air and the potential for an occult perforation, the patient was stabilized and transferred the same day to a tertiary center. Endoscopic evaluation was performed after transfer and revealed only superficial rectal mucosal abrasion, with no visible full‐thickness defect. As he remained hemodynamically stable, had no peritoneal signs, and showed no endoscopic evidence of perforation, non‐operative management was elected. Conservative treatment then consisted of bowel rest, rectal lavage and irrigation, and broad‐spectrum antibiotics. No scrotal decompression was performed; the pneumoscrotum was managed with observation. During hospitalization, the patient had no fever, peritonitis, hemodynamic deterioration, or need for surgery. He was discharged in good condition. No follow‐up CT was available to document radiologic resolution.

## 3 Discussion

This case illustrates three points that we believe are clinically and radiologically important. First, serious internal injury can occur even when the patient is clothed and the air nozzle is not introduced into the anus. A focused high‐pressure jet can rapidly overcome sphincter resistance and produce abrupt rectal pressurization; the funnel‐shaped contour of the buttocks may further channel airflow toward the anal canal [4, 5, 14]. Experimental work has indicated that clothing offers only limited protection and primarily when the jet is applied at a distance from the anal region [8]. Our patient was wearing two layers of clothing at the time of injury, yet sustained widespread air dissection, directly relevant to occupational safety education.

Second, injury severity depends on the interplay between air pressure, exposure duration, nozzle‐to‐anus distance, integrity of the clothing barrier, resting sphincter tone, and the compliance of the rectum and colon. Experimental and clinical data suggest that bowel‐wall injury progresses sequentially, beginning with serosal damage and advancing to muscular and mucosal disruption as intraluminal pressure rises [3, 8]. The pressures generated by industrial compressed‐air systems greatly exceed the threshold for experimental colonic perforation; severe cases therefore typically present with peritoneal signs, pneumoperitoneum, and visible wall defects [2, 3, 15].

Third, our case differs from the majority of published reports because no persistent full‐thickness rectal wall defect was demonstrable on either routine CT or endoscopy, despite striking extraluminal air. Several non‐mutually exclusive mechanisms may explain this discrepancy, but the most cautious interpretation is air escape through an occult sub‐endoscopic mucosal breach or transiently sealed microperforation. Such a breach could permit high‐pressure air to dissect into perirectal and retroperitoneal tissues while becoming too small, sealed, or decompressed to be identified on routine CT or endoscopy [5, 6]. Therefore, the absence of a visible persistent full‐thickness defect should not be interpreted as proof that no luminal communication occurred; conversely, it also should not be equated automatically with gross fecal perforation in a stable patient without contamination.

The compartmental distribution of air supports a retroperitoneal and fascial‐plane dissection model. A low extraperitoneal rectal breach can introduce air into the perirectal tissues, from which it may spread cephalad and laterally through the retroperitoneum and along the abdominopelvic fascial planes [7, 13]. The scrotum communicates with the superficial fascia of the perineum and lower abdominal wall, and retroperitoneal air may track inferiorly through the perinephric and anterior pararenal spaces into the inguinal canal and down the spermatic cord, accounting for pneumoscrotum [7, 13]. Air may also ascend along paraspinal pathways and enter the epidural space through the neural foramina, producing pneumorrhachis [9, 11]. Extradural pneumorrhachis without neurological deficit, as in our patient, is generally benign and resolves with treatment of the underlying source, whereas intradural air suggests more severe trauma and warrants further investigation [10, 14].

From a radiological standpoint, this case underscores the central role of multidetector CT in the emergency evaluation of anorectal barotrauma. Physical examination alone may underestimate the severity of injury because the anal verge and distal rectal mucosa often appear preserved and rectal bleeding may be absent. CT rapidly demonstrates the presence, extent, and compartmental distribution of extraluminal gas and, crucially, identifies findings that shift management toward surgery: a discrete bowel‐wall defect, pneumoperitoneum with peritoneal contamination, large fluid collections, extraluminal fecal material, active bleeding, or signs of bowel ischemia [7, 13]. In their absence, and provided the patient remains clinically stable, a non‐operative approach may be reasonable. In our patient, because no water‐soluble contrast leak study was performed, the decision for conservative treatment was based on the combined absence of peritonitis, hemodynamic instability, large fluid collection, fecal contamination, active bleeding, or clinical deterioration rather than on definitive exclusion of all luminal communication.

Management of compressed‐air colorectal injury must therefore be individualized. Patients with hemodynamic instability, peritonitis, tension pneumoperitoneum, definite perforation, or fecal contamination require urgent surgical evaluation, and percutaneous decompression may occasionally be needed for tension pneumoperitoneum. Reported surgical strategies include primary repair, resection, and fecal diversion, depending on the size and location of the defect and the degree of contamination [1, 2, 3]. Conversely, carefully selected patients without peritonitis, without a demonstrable persistent full‐thickness rectal wall defect, and without radiologic evidence of fecal contamination may be candidates for conservative therapy [4, 6]. Our patient met these criteria and recovered with bowel rest, lavage, irrigation, and antibiotics.

The novelty of the present case lies in the combination of extensive multicompartmental air dissection, pneumoscrotum, and pneumorrhachis after perineal compressed‐air injury without a demonstrable persistent full‐thickness rectal defect on routine CT or endoscopy. Most prior reports have emphasized frank perforation, fecal peritonitis, and operative management [3, 12, 14], and the few accounts of pneumoscrotum after perineal trauma or extraperitoneal perforation have generally lacked concurrent pneumorrhachis [4, 13, 15]. By contrast, our case represents an imaging‐dominant presentation in which CT showed dramatic extraintestinal air spread while endoscopy revealed only mucosal abrasion. Recognizing this pattern is clinically important: misinterpreting striking imaging findings as equivalent to gross perforation may lead to unnecessary surgery, while overreliance on negative endoscopy may produce false reassurance. The appropriate strategy integrates mechanism, physical examination, CT, endoscopy, and serial clinical assessment.

This case has clear occupational‐safety implications. Compressed‐air devices are ubiquitous in industrial environments, and their misuse, even briefly or through clothing, can cause severe and potentially fatal internal injury. Targeted education on the dangers of directing compressed air toward any part of the body, particularly the perineum, is essential. Several limitations should nonetheless be acknowledged. This is a single‐patient case report, so the conclusions cannot be generalized; the exact site of mucosal injury could not be definitively identified on either CT or endoscopy; no water‐soluble rectal contrast leak study was performed; the intraluminal air pressure at the moment of insult could not be measured; no follow‐up CT was available to document radiologic resolution; and long‐term follow‐up beyond hospital discharge was limited.

## 4 Conclusion

High‐pressure perineal compressed‐air injury may produce extensive multicompartmental extraluminal air dissemination, including pneumoscrotum and pneumorrhachis, even when routine CT and endoscopy do not demonstrate a persistent full‐thickness rectal defect. In this setting, an occult sub‐endoscopic mucosal breach or transiently sealed microperforation remains the most cautious explanation. CT is central for mapping air distribution and detecting features that mandate surgery. Conservative management may be considered only in carefully selected, hemodynamically stable patients without peritonitis, fecal contamination, large fluid collection, active bleeding, or clinical deterioration.

## Author Contributions

**Vuong Hoang Trung Le:** investigation, data curation, writing – review and editing. **Van Trung Hoang:** conceptualization, investigation, visualization, writing – review and editing, writing – original draft. **Vichit Chansomphou:** investigation, writing – review and editing, visualization. **The Huan Hoang:** investigation, data curation, writing – review and editing.

## Funding

The authors have nothing to report.

## Ethics Statement

This case report was prepared retrospectively after completion of clinical care. Institutional ethics approval was waived in accordance with local regulations governing single‐patient case reports.

## Consent

Written informed consent was obtained from the patient for publication of the clinical details and anonymized imaging findings.

## Conflicts of Interest

The authors declare no conflicts of interest.

## Data Availability

All relevant clinical and imaging data are included within this article. Additional data are available from the corresponding author upon reasonable request, subject to patient confidentiality.
